# A test of the ecological valence theory of color preference, the case of Arabic

**DOI:** 10.3389/fpsyg.2022.1010108

**Published:** 2022-12-22

**Authors:** Abdulrahman S. Al-Rasheed, Anna Franklin, John Maule

**Affiliations:** ^1^Department of Psychology, King Saud University, Riyadh, Saudi Arabia; ^2^The Sussex Colour Group, The School of Psychology, University of Sussex, Brighton, United Kingdom

**Keywords:** visual perception, color, preference, ecological valence theory, color preferences

## Abstract

Humans have systematic and reliable color preferences. The dominant account of color preference is that individuals like some colors more than others due to the valence of objects that they associate with colors (Ecological Valence Theory). In support of this theory, Palmer and Schloss show that the average valence of objects associated with a color, when weighted (the WAVE), explains up to 80% of the variation in color preference for adults from the United States (US). Here we investigate whether Ecological Valence Theory can account for the color preferences of female and male adults from Saudi Arabia to test how well the theory generalizes across cultures and how well it accounts for sex differences in color preference. We also extend the investigation of EVT by investigating whether abstract concept associations as well as object associations can account for preference. Saudi adults’ color preferences, color object and concept associations, and association valence ratings were collected, and the WAVE was computed and correlated with preference ratings. The WAVE accounted for no more than half of the variance in Saudi color preferences, although there was some degree of sex specificity in the relationship of the WAVE and color preference. Adding abstract concept associations did not account for more variance than object associations alone, but the number of abstract concept associations did account for a significant amount of the variance in color preference for females, but not males. The findings converge with other cross-cultural studies in suggesting that the success of EVT in accounting for color preference varies across cultures and indicates that additional factors other than color associations are likely also at play.

## 1 Introduction

Decades of research has established that humans have systematic and reliable color preferences (e.g., Guilford and Smith, 1959; McManus et al., 1981; Hurlbert and Ling, 2007; Palmer and Schloss, 2010). Common patterns of color preference found in studies across the years, such as a preference for blue and a dislike of chartreuse, have also led to claims that certain aspects of color preference are “universal” (Palmer et al., 2013; Hurlbert and Owen, 2015). There has been concerted effort to explain why some colors appear to be liked more than others when shown devoid of context, and various models have been proposed (Hurlbert and Ling, 2007; Palmer and Schloss, 2010). There has also been examination of, and theorizing about sex differences in color preference (Ou et al., 2004a,b; Hurlbert and Ling, 2007; Palmer and Schloss, 2010), and exploration of how color preferences vary with culture (e.g., Taylor et al., 2013; Sorokowski et al., 2014; Al-Rasheed, 2015). The scientific investigation of color preference provides a simple way to measure the degree to which an affective response to a simple visual stimulus is universally constrained and the degree to which it is shaped by experience, tapping into debates about cultural relativity of perceptual experience (e.g., Bremner et al., 2016) and the nature of aesthetics (e.g., Palmer et al., 2013).

Although several accounts of color preference have been proposed over the years (e.g., Hurlbert and Ling, 2007; Franklin et al., 2010; Palmer and Schloss, 2010; Yokosawa et al., 2016), possibly the most dominant account is Ecological Valence Theory (EVT, Palmer and Schloss, 2010). EVT contends that color preferences are the result of an evolutionary process that steers individuals toward beneficial objects and away from harmful ones. The degree to which colors are associated with advantageous or disadvantageous objects or items determines how preferred they are when viewed in the abstract (i.e., when not applied to any object, such as a patch viewed on a screen). This theory is proposed to account for the “universal” adult preference for blue hues (through their association with, e.g., clear skies, clean water) and red hues (e.g., ripe berries), as well as the low preference for browns, olives, and chartreuse (e.g., faces, rotting food). The theory also contends that these universal ecological valences can be influenced by color and object associations learned in everyday life (e.g., associating red with your preferred football team). Color preference, therefore, emerges as a result of the unconscious integration of all of these associations.

To test the EVT hypothesis, Palmer and Schloss devised the weighted affective valence estimate (WAVE) model. This model calculates the average affective valence of people’s responses to objects associated with each color, weighted by the strength of association with that color. The WAVE for a given color is the result of three separate tasks: an object description task in which participants name objects associated with the specific color; a color object matching task in which the participants rate the strength of association between the color and named objects; and an object valence task in which participants rate the valence of the named objects. Palmer and Schloss (2010) also compared EVT to three other models that have attempted to predict color preference; the cone-opponent model which summarizes color preference in terms of weights on the cone-opponent mechanisms (Hurlbert and Ling, 2007; Ling and Hurlbert, 2009), a model derived from their participants’ ratings of color appearance, and color-emotion model proposed that color preference triggered when strong feelings are evoked by certain colors (Ou et al., 2004a,b). EVT was found to outperform the other three models, accounting for 80% of the variance in color preference, compared to 37% for the cone-opponent model, 60% for the color appearance model, and 55% for the color-emotion model.

That the valence of object associations with color can account for 80% of the variance in color preferences of US adults is striking. Note that the correlations are done on a group level and explain the average pattern of color preference: subsequent research has established that at an individual level the EVT performs substantially less well (Palmer and Schloss, 2010). Nevertheless, the demonstration of the explanatory power of EVT was further demonstrated by Taylor and Franklin (2012) who showed that the WAVE could account for 61% of variance in the average color preferences of British observers. In addition, they found that the WAVE was a better fit for male preferences (74% shared variance) than female (45% shared variance), which they suggested may reflect sex differences in the tendency to associate color with concrete objects and abstract conceptual/symbolic associations (which were excluded from Palmer and Schloss’ study and their WAVE calculation). Taylor and Franklin also found that the number of objects associated with a color was a good predictor of color preference–colors which were associated with fewer objects tended to be more preferred, potentially suggesting that the role of valence might be overestimated in EVT.

There have been just a few other studies which have investigated how EVT generalizes to other cultures, and WAVE has not performed as well outside of Western cultural contexts. An investigation of EVT in Japan found that the WAVE could account for 37% of the variance in color preference (Yokosawa et al., 2016)–substantially less than the 80% originally reported by Palmer and Schloss (2010) for their US sample. When Taylor et al. (2013) tested the color-object associations and color preference of the Himba (a non-industrialized indigenous group in northern Namibia) they found that WAVE was a relatively poor predictor of color preference, accounting for only 23% of the variance in color preference (a simple model of saturation was the best predictor). The relationship was also opposite in direction to that predicted by EVT: colors that Himba adults associated with negatively valenced objects were more preferred.

One possible interpretation for the variability of the success of EVT to account for color preference is that the contribution of abstract conceptual (i.e., symbolic) color associations, which were excluded in the original EVT studies, is more pivotal to color preferences in some cultures compared to others. The success of EVT in accounting for color preference may improve for some cultures if abstract conceptual associations are included. Support for this hypothesis was provided in a study which included such associations (e.g., white and peace) in the computation of the WAVE and related this to color preference for US and Chinese samples (unpublished study discussed in Schloss and Palmer, 2017). The WAVEs based on symbolic associations explained more variance in Chinese color preference (54%) than the WAVEs based on object associations (25%). In contrast, the pattern for the US participants showed the opposite, symbolic-WAVEs explained much less variance in color preference (34%) than the object-WAVES did (80%).

The current study further investigates the generalisability of EVT across non-western cultures and investigates whether it can account for color preferences in Saudi Arabia. A prior study of Saudi color preference identified notable differences in color preferences for Saudi and other cultures: for example, Saudi females did not have the characteristic peak in preference for blueish hues (Al-Rasheed, 2015). The reason for this cultural variation in color preference is unclear. EVT would propose that such cultural differences in color preference is accounted for by cultural differences in the types of objects associated with colors and/or in the valence of those object associations. Arabic culture (specifically Saudi culture, as is investigated in the present research), has plenty of differences from Western culture. Two sources of variation could potentially play a critical role in color preference, providing a strong test of the generalizability of EVT. First, the arid desert climate means the local natural environment is dominated by reds and oranges, with relatively little vegetation [see Webster et al. (2007) for a comparison of lush and arid natural statistics in India], and lifestyles are typically city-based. Second, Saudi lifestyles involve greater segregation between men and women, particularly in the use of public spaces (e.g., at university), and so the associations between colors and objects for males and females may differ more in Saudi culture compared to cultural contexts in which men and women are less segregated.

The current study investigates whether EVT can account for Saudi color preferences and sex differences in these preferences. We also extend Palmer and Schloss’ original investigation of EVT to include abstract conceptual color associations and not just object associations with color. Abstract concept associations are traditionally omitted from EVT, and yet color is highly conceptual and symbolic (Mukherjee et al., 2022) and as we see in Schloss and Palmer (2017), the contribution of abstract conceptual associations to color preference may be particularly important in some cultures. Here, we include abstract concept associations (referred hereafter as concept associations) and analyses their contribution to color preference both separately and when combined with object associations. This enables us to clarify the role of objects and concepts in color preference, and also provides the first test of Taylor and Franklin’s hypothesis that EVT works less well for females because females weight conceptual color associations more strongly than males. We also further investigate Taylor and Franklin’s finding that the number of associations with a color also relates to color preference irrespective of valence. Overall, the current study aims to establish the generalisability and limits of the explanatory power of EVT.

## 2 Materials and methods

### 2.1 Participants

Participants were 409 Saudi undergraduates (204 females, 205 males, mean age = 21 years, range 18–27 years) recruited from the student population of the University of King Saud, Riyadh, Saudi Arabia. All passed the City University Test (Fletcher, 1980), indicating no color vision deficiency. Participants were assigned to one of four different tasks such that male or female sample sizes for each task ranged from *N* = 49–55 (mean age 21 years for each task). Male participants were tested by the first author and female participants by female research staff.

### 2.2 Stimuli and set-up

The stimuli were 24 colors based on the “saturated” (S), “light” (L), and “dark” (D) sets of the Berkeley Color Project (Palmer and Schloss, 2010). Within each set there were eight hues: (R), orange (O), yellow (Y), chartreuse (H), green (G), cyan (C), blue (B), and purple (P). The 8 “muted” stimuli used by Palmer and Schloss were not included in the current study because the preference curve and WAVEs for that set were similar to the “light” set in Palmer and Schloss’ original investigation. The rendered colors were largely identical to Palmer and Schloss’ study with the exception of five of the colors (SR, SO, LO, SY, SP, and LY) which were reduced slightly in saturation to fit into the gamut of the monitor. See Table 1 in the SI for stimulus co-ordinates. Stimuli were rendered on a 17-inch CRT monitor and were calibrated for each display using a ColorCal MKII Colorimeter (Cambridge Research Systems), measuring the center of the display. There was no noticeable variation associated with participants’ viewing angle (see Section “Task design and procedure,” below) in the appearance of the colors when presented on the calibrated displays.

**TABLE 1:** Coordinates in Munsell color space and the CIE (1932) Y,x,y chromaticity coordinates for the twenty-four colors and the gray background and white point of the monitor.

Color-code		Munsell			CIE co-ordinates		
		Hue	Value	Chroma	*Y*	*x*	*y*
St1	Saturated Red (SR)	5R	5	14	19.27	0.534	0.316
St2	Light Red (LR)	5R	7	8	41.99	0.407	0.326
St3	Dark Red (DR)	5R	3	8	6.39	0.506	0.311
St4	Saturated Orange (SO)	5YR	6.5	12	35.30	0.511	0.410
St5	Light Orange (LO)	5YR	8	5	57.62	0.384	0.359
St6	Dark Orange (DO)	5YR	3.5	6	8.78	0.482	0.389
St7	Saturated Yellow (SY)	5Y	8	11	57.62	0.447	0.469
St8	Light Yellow (LY)	5Y	8.5	5.5	66.69	0.382	0.402
St9	Dark Yellow (DY)	5Y	5	6.5	19.27	0.437	0.450
St10	Saturated Chartreuse (SH)	5GY	8	11	57.62	0.387	0.504
St11	Light chartreuse (LH)	5GY	8.5	6	66.69	0.357	0.420
St12	Dark chartreuse (DH)	5GY	4.5	6	15.19	0.369	0.474
St13	Saturated Green (SG)	3.75G	6.5	11.5	35.30	0.252	0.448
St14	Light Green (LG)	3.75G	7.75	6.25	53.41	0.287	0.381
St15	Dark Green (DG)	3.75G	3.75	6.25	10.17	0.260	0.418
St16	Saturated Cyan (SC)	5BG	7	9	41.99	0.226	0.335
St17	Light Cyan (LC)	5BG	8	5	57.62	0.267	0.330
St18	Dark Cyan (DC)	5BG	4	5	11.70	0.233	0.324
St19	Saturated Blue (SB)	10B	6	10	29.30	0.200	0.230
St20	Light Blue (LB)	10B	7.5	5.5	49.41	0.255	0.278
St21	Dark Blue (DB)	10B	3.5	5.5	8.78	0.211	0.235
St22	Saturated Purple (SP)	5P	4.5	7	15.19	0.289	0.231
St23	Light Purple (LP)	5P	7	9	41.99	0.290	0.242
St24	Dark Purple (DP)	5P	3	9	6.39	0.280	0.181
Gray background		–	–	–	19.26	0.312	0.318
White point		–	–	–	93.26	0.297	0.32

### 2.3 Task design and procedure

Participants took part in one of four tasks as in Palmer and Schloss (2010). Task instructions were translated by a native Arabic speaker with native-like competence in English and delivered in Arabic by native Arabic speakers.

#### 2.3.1 Color preference task

The 24-computer rendered color stimuli were presented as squares (5 × 5 cm) one at a time in a random order on a gray background until the participant made their judgement, separated by a 500 ms inter-trial interval. Participants were asked to rate each one using a line-mark slider scale anchored by the terms “not at all” and “very much” at either end.

#### 2.3.2 Association task

Participants were shown each stimulus on a gray background in a random order and were asked to list of objects or concepts that they associate with the color presented on a screen. Participants were tested in 20 groups, with 5 participants in each group, seated in a half circle facing the screen and each made their own list without conferring. Participants were instructed to write down as many objects or concepts as they can think of that specific color, and that they should list both objects and abstract concepts (i.e. love, peace, and hate, etc.), but not personal associations (i.e., not the color of their bedroom walls), and not to list objects that could be any color (i.e., clothing, cars, etc.). There was no time limit. If an object description failed to meet the above criteria or was not cited by more than one participant for any of the colors, it was removed from the analysis. We allowed object associations which could be a small number of particular, non-arbitrary colors (e.g., traffic light), since this association is still expected to be consistent across different observers.

#### 2.3.3 Valence task

Participants were visually presented with the terms listed in the Association Task, one at a time and in a random order, and were asked to rate the term according to its’ valence using the rating scale from the Color Preference Task but with “negative” and “positive” end points.

#### 2.3.4 Color-term matching task

Participants were shown each color square with each of the terms associated with that specific color, one at a time, in a random order. Participants were asked to rate how closely the term and color matched using a line rating scale with “very poorly” and “very well” as opposite end points.

## 3 Results

**FIGURE 1:**
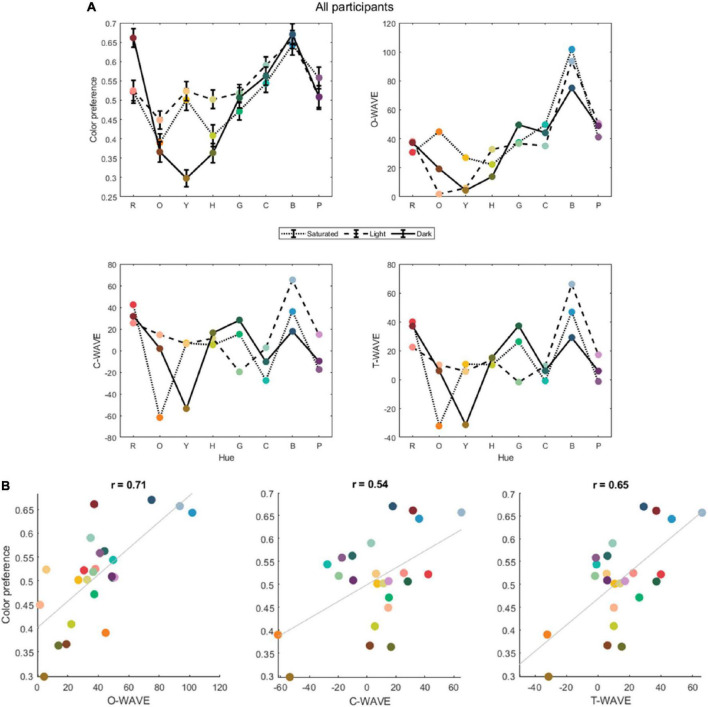
**(A)** The average color preference rating and WAVEs for saturated (S), light (L), dark (D) versions of 8 hues, for object associations (O-WAVE), concept associations (C-WAVE) and both together (T-WAVE), for all participants. **(B)** The correlation plots between color preference and the three WAVE types: O-Wave, C-Wave, and T-Wave for all participants. Points are filled with color indicative of their appearance in the experiments, although note there are differences between display devices and so this is only an approximate rendering.

Palmer and Schloss (2010) theorized that preference for a given color is determined by the average affective valence of the objects which are associated with that color, when averaged across persons. We tested this hypothesis by correlating the average color preferences for each of the 24 colors (Figure 1), with the corresponding weighted affective valence estimate for associations with the colors (WAVE). Whereas Palmer and Schloss calculated the WAVE only for associated objects, in the current study we also solicited concept associations. We present the results for when only object associations are included (O-WAVE), for only concept associations (C-WAVE), and then for both together (T-WAVE). The calculation of the WAVEs for the 24 colors was based on the analysis of the results of the association, color-term matching, and association valence tasks. The WAVE was calculated according to the formula of Palmer and Schloss (2010): $W_{C}=\frac{1}{n_{c}}\,\sum_{o=1}^{n_{c}}w_{c\,o}\,v_{o}$, where w_co_ is the average color-term match value for each term and its associated color, *v*_o_ is the average preference rating for each term, and *n*_c_ is the number of terms associated with each color (Table 2 in the appendix for more details). Responses on the rating scales were initially coded in 1-unit increments from −200 to +200. Color preference and the association task ratings were subsequently transformed to be in the range 0–1 (e.g., Figure 1) for calculation of the WAVE. Therefore, WAVE ranges from −200 to +200.

**TABLE 2:** For each color, the associated Arabic objects, the English translated, the average color preference rating, the average match between the color and its associated objects, and the average valence for the objects, for male, female, and overall.

Color	Arabic object description	English translation	Color preference rating			Color-object match			Object rating		
			Male	Female	Overall	Male	Female	Overall	Male	Female	Overall
SR	دم	Blood	27.38	–9.95	8.72	102.29	64.06	83.18	–59.92	–75.45	–67.69
SR	حب	Love	27.38	–9.95	8.72	105.47	39.52	72.50	144.90	136.62	140.76
SR	وردة	Flower	27.38	–9.95	8.72	119.98	85.56	102.77	119.14	115.79	117.47
SR	نار	Fire	27.38	–9.95	8.72	12.35	38.26	25.31	9.16	–17.96	–4.40
SR	اشارة مرور	Traffic light	27.38	–9.95	8.72	119.33	86.72	103.03	13.43	–21.30	–3.94
SR	فراولة	Strawberry	27.38	–9.95	8.72	120.29	78.06	99.18	99.78	108.06	103.92
SR	أحمر شفاه	Lipstick	27.38	–9.95	8.72	96.98	95.9	96.44	–21.55	86.75	32.60
SR	قلب	Heart	27.38	–9.95	8.72	116.00	78.06	97.03	126.80	122.98	124.89
SR	ملفت	Attractive	27.38	–9.95	8.72	79.67	125.08	102.37	49.41	59.42	54.41
SR	فاقع	Bright	27.38	–9.95	8.72	77.35	125.26	101.31	–45.06	–67.45	–56.26
SR	كرز	Cherry	27.38	–9.95	8.72	49.59	25.42	37.50	80.71	59.15	69.93
SR	القوة	Strength	27.38	–9.95	8.72	–14.27	–20.84	–17.56	129.41	151.32	140.36
SR	إزعاج	Annoying/ed	27.38	–9.95	8.72	3.33	63.26	33.30	–142.29	–122.96	–132.62
SR	طماط	Tomato	27.38	–9.95	8.72	118.33	106.18	112.26	14.71	–6.79	3.96
LR	ورد	Flowers	–38.02	57.65	9.82	36.35	49.84	43.10	117.04	124.11	120.58
LR	انثوي	Feminine	–38.02	57.65	9.82	94.20	94.8	94.50	–7.78	93.06	42.64
LR	مكياج	Makeup	–38.02	57.65	9.82	85.00	122.6	103.80	–47.43	84.72	18.64
LR	نعومة	Daintiness	–38.02	57.65	9.82	70.96	90.76	80.86	38.88	140.62	89.75
LR	أحمر خدود	Blusher	–38.02	57.65	9.82	44.84	110.64	77.74	–5.69	80.74	37.52
LR	هدوء	Calm	–38.02	57.65	9.82	–3.25	–33.5	–18.38	148.12	136.23	142.17
LR	طفولي	Childish	–38.02	57.65	9.82	15.59	7.36	11.47	30.06	84.30	57.18
LR	أحمر شفاه	Lipstick	–38.02	57.65	9.82	44.86	76.86	60.86	–21.55	86.75	32.60
LR	ناعم	Smooth	–38.02	57.65	9.82	55.45	83.6	69.53	55.96	130.21	93.08
LR	الغروب	Sunset	–38.02	57.65	9.82	–37.61	–54.32	–45.96	86.71	101.28	94.00
LR	زهرة	Rose	–38.02	57.65	9.82	29.45	47.32	38.39	98.43	89.66	94.04
LR	لطافة	Nice	–38.02	57.65	9.82	56.02	81.7	68.86	122.69	154.83	138.76
LR	بشرة	Skin	–38.02	57.65	9.82	–19.69	–23.4	–21.54	57.88	82.49	70.18
LR	جمال	Beauty	–38.02	57.65	9.82	11.94	50.98	31.46	121.53	133.15	127.34
DR	دم	Blood	51.14	77.91	64.52	135.08	126.74	130.91	–59.92	–75.45	–67.69
DR	أحمر شفاه	Lipstick	51.14	77.91	64.52	121.43	125.28	123.36	–18.96	95.32	38.18
DR	ورد	Flowers	51.14	77.91	64.52	118.88	105.84	112.36	117.04	124.11	120.58
DR	حب	Love	51.14	77.91	64.52	95.98	24.84	60.41	144.90	136.62	140.76
DR	قلب	Heart	51.14	77.91	64.52	108.63	87.48	98.05	126.80	122.98	124.89
DR	أحمر شفاه	Lipstick	51.14	77.91	64.52	89.39	122.96	106.18	–21.55	86.75	32.60
DR	طماطم	Tomato	51.14	77.91	64.52	89.29	–18.56	35.37	14.78	18.47	16.62
DR	تفاح	Apple	51.14	77.91	64.52	77.98	29.94	53.96	68.67	55.23	61.95
DR	جمال	Beauty	51.14	77.91	64.52	16.35	12.34	14.35	121.53	133.15	127.34
DR	توت	Berries	51.14	77.91	64.52	66.57	8.88	37.72	102.02	84.96	93.49
DR	عنب	Grape	51.14	77.91	64.52	8.41	–67.18	–29.38	79.45	75.91	77.68
DR	شمس	Sun	51.14	77.91	64.52	–137.88	−153	–145.44	63.14	33.19	48.17
DR	فراولة	Strawberry	51.14	77.91	64.52	93.94	17.3	55.62	99.78	108.06	103.92
DR	كرز	Cherry	51.14	77.91	64.52	73.90	83.06	78.48	80.71	59.15	69.93
DR	لحم	Meat	51.14	77.91	64.52	92.61	84.48	88.54	81.76	16.06	48.91
SO	برتقال	Orange	–22.44	–65.62	–44.03	145.53	72.36	108.94	73.02	59.87	66.44
SO	الغروب	Sunset	–22.44	–65.62	–44.03	50.47	35.84	43.16	86.71	101.28	94.00
SO	نار	Fire	–22.44	–65.62	–44.03	24.69	–7.34	8.67	9.16	–17.96	–4.40
SO	شمس	Sun	–22.44	–65.62	–44.03	25.37	–26.42	–0.52	63.14	33.19	48.17
SO	عصير	Juice	–22.44	–65.62	–44.03	82.02	129.42	105.72	104.49	69.42	86.95
SO	يوسف أفندي	Mandarin	–22.44	–65.62	–44.03	104.35	146.56	125.46	72.22	49.00	60.61
SO	الشروق	Sunrise	–22.44	–65.62	–44.03	4.06	9.04	6.55	118.45	144.17	131.31
SO	سعادة	Happiness	–22.44	–65.62	–44.03	–60.45	–75.04	–67.75	146.59	144.92	145.76
LO	جلد	Skin	–24.32	–16.58	–20.45	80.06	35.72	57.89	57.88	82.49	70.18
LO	جلد	Skin	–24.32	–16.58	–20.45	89.94	45.94	67.94	5.92	–13.96	–4.02
LO	مشمش	Apricot	–24.32	–16.58	–20.45	3.84	58.2	31.02	15.24	7.75	11.50
LO	برتقال	Orange	–24.32	–16.58	–20.45	–109.35	–106.54	–107.95	73.02	59.87	66.44
LO	فاتح	Light (in color)	–24.32	–16.58	–20.45	65.92	65.94	65.93	60.18	76.21	68.20
LO	خوخ	Peach	–24.32	–16.58	–20.45	–40.57	31.2	–4.68	51.06	43.34	47.20
LO	الهدوء	Calm	–24.32	–16.58	–20.45	–18.78	–16.12	–17.45	148.12	136.23	142.17
LO	مكياج	Makeup	–24.32	–16.58	–20.45	82.31	75.84	79.08	–47.43	84.72	18.64
LO	تراب	Soil	–24.32	–16.58	–20.45	15.69	33.46	24.57	–5.10	–20.21	–12.65
LO	رمل غبار	Sand Dust	–24.32	–16.58	–20.45	43.96	47.18	45.57	–115.55	–137.58	–126.57
DO	خشب	Wood	–17.44	–89.69	–53.57	100.33	21.24	60.79	40.55	58.57	49.56
DO	طين	Mud	–17.44	–89.69	–53.57	108.25	6.3	57.28	–29.69	–62.08	–45.88
DO	عود	Oud	–17.44	–89.69	–53.57	78.94	30.42	54.68	117.20	77.02	97.11
DO	براز	Feces	–17.44	–89.69	–53.57	87.14	–6.22	40.46	–154.92	–158.47	–156.70
DO	شكلاته	Chocolate	–17.44	–89.69	–53.57	123.80	81.5	102.65	88.82	89.09	88.96
DO	جذع النخل	Palm tree trunk	–17.44	–89.69	–53.57	49.43	–18.8	15.32	4.88	–10.00	–2.56
DO	باب	Door	–17.44	–89.69	–53.57	63.53	79.38	71.45	42.39	36.13	39.26
DO	بخور	Incense	–17.44	–89.69	–53.57	15.88	23.56	19.72	110.22	129.77	120.00
DO	حطب	Wood	–17.44	–89.69	–53.57	61.43	24.04	42.74	56.80	58.34	57.57
DO	كئابة	Depression	–17.44	–89.69	–53.57	–1.73	25.94	12.11	–109.18	–135.30	–122.24
DO	جبل	Mountain	–17.44	–89.69	–53.57	7.86	3.46	5.66	71.61	50.87	61.24
SY	شمس	Sun	–21.56	22.53	0.48	105.88	83.1	94.49	63.14	33.19	48.17
SY	ليمون	Lemon	–21.56	22.53	0.48	97.22	60.86	79.04	52.53	80.49	66.51
SY	موز	Banana	–21.56	22.53	0.48	106.84	42.42	74.63	54.65	17.47	36.06
SY	بول	Urine	–21.56	22.53	0.48	68.65	24.1	46.37	–128.71	–147.60	–138.16
SY	ورد	Flowers	–21.56	22.53	0.48	58.02	48.76	53.39	117.04	124.11	120.58
SY	برتقال	Orange	–21.56	22.53	0.48	–7.04	–37.56	–22.30	73.02	59.87	66.44
SY	الشروق	Sunrays	–21.56	22.53	0.48	72.98	58.74	65.86	118.45	144.17	131.31
SY	ذهب	Gold	–21.56	22.53	0.48	97.24	51.94	74.59	120.29	107.08	113.68
SY	منقى	Purified	–21.56	22.53	0.48	51.59	62.24	56.91	55.80	67.47	61.63
SY	السعادة	Happiness	–21.56	22.53	0.48	–0.43	19	9.28	162.69	167.09	164.89
SY	حرارة	Heat	–21.56	22.53	0.48	–3.16	24.58	10.71	–58.53	–93.55	–76.04
SY		soccer club	–21.56	22.53	0.48	85.33	54.46	69.90	−	−	−
SY	القوة	Strength	–21.56	22.53	0.48	–42.55	–6.1	–24.32	129.41	151.32	140.36
LY	شمس	Sun	–1.4	19.91	9.25	21.82	30.78	26.30	63.14	33.19	48.17
LY	ليمون	Lemon	–1.4	19.91	9.25	8.16	–11.04	–1.44	52.53	80.49	66.51
LY	موز	Banana	–1.4	19.91	9.25	24.35	24.98	24.67	54.65	17.47	36.06
LY	هاديء	Calm	–1.4	19.91	9.25	–31.92	10.02	–10.95	148.12	136.23	142.17
LY	شمام	Melon	–1.4	19.91	9.25	79.92	85.64	82.78	20.51	–0.32	10.09
LY	ضوء	Light	–1.4	19.91	9.25	46.90	69.4	58.15	69.10	70.68	69.89
LY	صحراء	Desert	–1.4	19.91	9.25	92.92	55.38	74.15	43.96	–44.00	–0.02
LY	ترب	Soil	–1.4	19.91	9.25	64.14	1.42	32.78	–5.10	–20.21	–12.65
LY	غبار	Dust	–1.4	19.91	9.25	73.35	11.46	42.41	–124.80	–128.09	–126.45
LY	رمل	Sand	–1.4	19.91	9.25	77.51	23.36	50.43	36.53	–1.60	17.46
LY	حليب موز	Banana milk	–1.4	19.91	9.25	41.86	77.68	59.77	23.80	–72.66	–24.43
LY	راحة	Rest	–1.4	19.91	9.25	–23.76	–1.04	–12.40	154.31	172.28	163.29
LY	شاطيء	Beach	–1.4	19.91	9.25	23.63	–42.6	–9.49	112.63	140.64	126.64
DY	ذهب	Gold	–68.5	–93.44	–80.97	4.00	–102.64	–49.32	120.29	107.08	113.68
DY	كركم	Turmeric	–68.5	–93.44	–80.97	54.27	64.94	59.61	–8.71	–19.72	–14.22
DY	ترب	Soil	–68.5	–93.44	–80.97	32.63	8.86	20.74	–3.71	–30.28	–17.00
DY	براز	Faces	–68.5	–93.44	–80.97	–5.27	24.48	9.60	–154.92	–158.47	–156.70
DY	ليمون	Lemon	–68.5	–93.44	–80.97	–88.20	–136.92	–112.56	52.53	80.49	66.51
DY	شمس	Sun	–68.5	–93.44	–80.97	–93.14	–137.66	–115.40	63.14	33.19	48.17
DY	قهوة	Coffee	–68.5	–93.44	–80.97	–0.73	55.84	27.56	88.18	132.21	110.20
DY	مميز	Special	–68.5	–93.44	–80.97	–34.20	–94.98	–64.59	130.06	132.43	131.25
DY	عود	Oud	–68.5	–93.44	–80.97	–10.57	–25.34	–17.95	117.20	77.02	97.11
DY	طين	Mud	–68.5	–93.44	–80.97	23.45	66.62	45.04	–29.69	–62.08	–45.88
DY	رمل	Sand	–68.5	–93.44	–80.97	16.47	–33.3	–8.41	36.53	–1.60	17.46
DY	زيت	Oil	–68.5	–93.44	–80.97	52.71	–39.56	6.57	–3.57	–47.49	–25.53
SH	شمس	Sun	–48.74	–24.60	–36.67	–4.86	–22.44	–13.65	63.14	33.19	48.17
SH	ليمون	Lemon	–48.74	–24.60	–36.67	85.76	54.36	70.06	52.53	80.49	66.51
SH	الشروق	Sunrays	–48.74	–24.60	–36.67	7.51	–12.18	–2.34	118.45	144.17	131.31
SH	تفاح أخضر	Green apple	–48.74	–24.60	–36.67	–69.20	–69.42	–69.31	53.41	31.60	42.51
SH	موز	Banana	–48.74	–24.60	–36.67	46.86	26.3	36.58	54.65	17.47	36.06
SH	مشرق	bright	–48.74	–24.60	–36.67	18.51	16.36	17.43	111.92	119.17	115.54
SH	الضوء	Lighting	–48.74	–24.60	–36.67	26.10	26.92	26.51	69.73	60.25	64.99
SH	نبات	Plant	–48.74	–24.60	–36.67	–35.96	–64.54	–50.25	82.94	95.79	89.37
SH	عنب	Grape	–48.74	–24.60	–36.67	–30.96	–104.96	–67.96	79.45	75.91	77.68
SH	كئابة	Depression	–48.74	–24.60	–36.67	–81.25	–74.6	–77.93	–109.18	–135.30	–122.24
SH	كمثرى	Pear	–48.74	–24.60	–36.67	45.39	46.82	46.11	46.82	24.58	35.70
LH	شمس	Sun	–21.04	22.58	0.77	17.90	25.02	21.46	52.18	24.77	38.48
LH	ليمون	Lemon	–21.04	22.58	0.77	42.53	80.68	61.60	52.53	80.49	66.51
LH	موز	Banana	–21.04	22.58	0.77	46.45	67.38	56.92	54.65	17.47	36.06
LH	الشروق	Sunrise	–21.04	22.58	0.77	–0.67	–6.54	–3.60	118.45	144.17	131.31
LH	الصباح	Morning	–21.04	22.58	0.77	–6.86	27.06	10.10	115.39	140.70	128.04
LH	إضاءة	Light	–21.04	22.58	0.77	42.29	43.62	42.96	72.59	69.49	71.04
LH	الراحة	Rest	–21.04	22.58	0.77	–57.29	16.02	–20.64	154.31	172.28	163.29
LH	الصيف	Summer	–21.04	22.58	0.77	31.57	81.6	56.58	0.57	–27.85	–13.64
LH	الصحراء	Desert	–21.04	22.58	0.77	27.16	–55.22	–14.03	37.71	–27.87	4.92
DH	زرع	Grass	–44.56	–64.84	–54.70	41.90	37.48	39.69	76.27	84.85	80.56
DH	غابة	Forest	–44.56	–64.84	–54.70	41.96	38.58	40.27	75.08	46.81	60.95
DH	أشجار	Trees	–44.56	–64.84	–54.70	46.55	56.22	51.38	83.12	119.21	101.16
DH	طحالب	Algae	–44.56	–64.84	–54.70	79.20	119.78	99.49	–104.24	–111.30	–107.77
DH	حديقة	Garden	–44.56	–64.84	–54.70	48.04	51.9	49.97	103.16	106.25	104.70
DH	طبيعة	Nature	–44.56	–64.84	–54.70	30.67	47.08	38.87	146.78	152.75	149.77
DH	كيوي	Kiwi	–44.56	–64.84	–54.70	121.33	97.4	109.37	33.59	17.94	25.77
DH	علم السعودية	Saudi Arabia flag	–44.56	–64.84	–54.70	–39.24	–40.52	–39.88	134.84	115.36	125.10
DH	عنب	Grapes	–44.56	–64.84	–54.70	–35.80	–89.92	–62.86	79.45	75.91	77.68
DH	زيت	Oil	–44.56	–64.84	–54.70	31.53	–43.24	–5.86	–3.57	–47.49	–25.53
DH	براز	Feces	–44.56	–64.84	–54.70	–58.80	−102	–80.40	–154.92	–158.47	–156.70
DH	كئابة	Depression	–44.56	–64.84	–54.70	–19.06	14.74	–2.16	–109.18	–135.30	–122.24
SG	نبات	Plant	3.02	–26.13	–11.55	85.76	30.84	58.30	82.94	95.79	89.37
SG	أشجار	Trees	3.02	–26.13	–11.55	87.37	38.74	63.06	83.12	119.21	101.16
SG	أوراق الشجر	Tree leaves	3.02	–26.13	–11.55	92.27	28.26	60.27	62.27	80.91	71.59
SG	علم السعودية	Saudi Arabia flag	3.02	–26.13	–11.55	87.76	32.72	60.24	134.84	115.36	125.10
SG	حديقة	Garden	3.02	–26.13	–11.55	89.33	30.04	59.69	103.16	106.25	104.70
SG	طبيعة	Nature	3.02	–26.13	–11.55	95.49	43.52	69.51	146.78	152.75	149.77
SG	ملعب	Stadium	3.02	–26.13	–11.55	101.14	73.84	87.49	52.35	–3.38	24.48
SG	خيار	Cucumber	3.02	–26.13	–11.55	69.92	5.38	37.65	50.61	44.77	47.69
SG	الربيع	Spring	3.02	–26.13	–11.55	61.92	28.26	45.09	143.82	137.11	140.46
SG	السلام	Peace	3.02	–26.13	–11.55	26.24	36.42	31.33	163.06	181.43	172.25
SG	استرخاء	Rest	3.02	–26.13	–11.55	19.45	–44.06	–12.30	143.06	174.53	158.79
SG	عشب	Grass	3.02	–26.13	–11.55	92.47	40	66.24	83.31	90.89	87.10
SG	طحالب	Algae	3.02	–26.13	–11.55	44.47	36.26	40.37	–104.24	–111.30	–107.77
SG	ضفدع	Frog	3.02	–26.13	–11.55	84.53	72.1	78.31	–53.98	–104.09	–79.04
SG	بطيخ	Watermelon	3.02	–26.13	–11.55	88.84	33.44	61.14	78.22	72.28	75.25
SG	هدوء	Calm	3.02	–26.13	–11.55	–21.57	–73.74	–47.65	148.12	136.23	142.17
SG	خضروات	Vegetables	3.02	–26.13	–11.55	86.16	60.88	73.52	55.27	41.98	48.62
LG	زرعش	Grass	11	3.64	7.32	18.59	–25.68	–3.55	83.31	90.89	87.10
LG	فرح	Happiness	11	3.64	7.32	–23.57	–62.18	–42.87	161.31	165.47	163.39
LG	عنب	Grapes	11	3.64	7.32	–22.29	–64.88	–43.59	79.45	75.91	77.68
LG	لطيف	Nice	11	3.64	7.32	–8.53	–20.84	–14.68	145.94	146.81	146.38
LG	طبيعة	Nature	11	3.64	7.32	28.57	11.14	19.85	146.78	152.75	149.77
LG	نعناع	Mint	11	3.64	7.32	18.49	–7.14	5.68	135.06	109.00	122.03
LG	بحر	Sea	11	3.64	7.32	–84.80	–135.52	–110.16	104.71	135.38	120.05
LG	خيار	Cucumber	11	3.64	7.32	–9.90	–22.28	–16.09	50.61	44.77	47.69
LG	الهدوء	Calm	11	3.64	7.32	–29.71	–67.22	–48.46	148.12	136.23	142.17
LG	راحة	Rest	11	3.64	7.32	–14.27	–35.1	–24.69	143.06	174.53	158.79
LG	أوراق الشجر	Tree leaves	11	3.64	7.32	10.06	–5.38	2.34	62.27	80.91	71.59
DG	علم السعودية	Saudi Arabia flag	11.62	–6.67	2.47	129.82	129	129.41	134.84	115.36	125.10
DG	زرع	Grass	11.62	–6.67	2.47	124.61	98.64	111.62	83.31	90.89	87.10
DG	أشجار	Trees	11.62	–6.67	2.47	129.04	114.98	122.01	83.12	119.21	101.16
DG	خيار	Cucumber	11.62	–6.67	2.47	118.57	95.62	107.09	50.61	44.77	47.69
DG	غابة	Forest	11.62	–6.67	2.47	103.00	101.26	102.13	75.08	46.81	60.95
DG	إشارة مرور	Traffic light	11.62	–6.67	2.47	71.71	2.2	36.95	34.06	–8.87	12.60
DG	طبيعة	Nature	11.62	–6.67	2.47	100.43	89.34	94.89	146.78	152.75	149.77
DG	أوراق الشجر	Tree leaves	11.62	–6.67	2.47	120.02	115.38	117.70	62.27	80.91	71.59
DG	حديقة	Garden	11.62	–6.67	2.47	100.10	90.44	95.27	103.16	106.25	104.70
DG	ملعب	Stadium	11.62	–6.67	2.47	103.73	71.9	87.81	52.35	–3.38	24.48
DG	نعناع	Mint	11.62	–6.67	2.47	120.90	80.9	100.90	135.06	109.00	122.03
DG	طحالب	Algae	11.62	–6.67	2.47	71.18	74.38	72.78	–104.24	–111.30	–107.77
DG	مزرعة	Farm	11.62	–6.67	2.47	97.49	84.22	90.86	88.27	107.85	98.06
DG	الراحة	Rest	11.62	–6.67	2.47	–10.25	–47.1	–28.68	154.31	172.28	163.29
DG	عشب	Grass	11.62	–6.67	2.47	35.31	49.9	42.61	–29.35	–26.79	–28.07
SC	سماء	Sky	35.12	–0.27	17.42	–10.53	–141.7	–76.11	133.67	172.49	153.08
SC	بحر	Sea	35.12	–0.27	17.42	17.20	–57.08	–19.94	104.71	135.38	120.05
SC	ملفت	Attractive	35.12	–0.27	17.42	11.63	23	17.31	49.41	59.42	54.41
SC	هاديء	Calm	35.12	–0.27	17.42	–0.20	–88.98	–44.59	148.12	136.23	142.17
SC	ماء	Water	35.12	–0.27	17.42	–7.94	–110.62	–59.28	159.90	169.32	164.61
SC	الفرح	Happiness	35.12	–0.27	17.42	–28.10	–71.8	–49.95	161.31	165.47	163.39
SC	مسبح	Pool	35.12	–0.27	17.42	29.88	–26.44	1.72	72.78	110.53	91.65
SC	استرخاء	Rest	35.12	–0.27	17.42	–17.59	–68.32	–42.95	143.88	161.55	152.71
LC	ماركة تفني	Tiffany brand	36.28	35.75	36.01	–8.86	111.42	51.28	–20.53	2.13	–9.20
LC	هدوء	Calm	36.28	35.75	36.01	16.59	21.56	19.07	148.12	136.23	142.17
LC	نهر	River	36.28	35.75	36.01	–22.63	–81.02	–51.82	111.47	124.87	118.17
LC	بحر	Sea	36.28	35.75	36.01	–27.14	–91.1	–59.12	104.71	135.38	120.05
LC	سماء	Sky	36.28	35.75	36.01	–24.88	–102.7	–63.79	133.67	172.49	153.08
LC	شاطيء	Beach	36.28	35.75	36.01	–27.82	–84.88	–56.35	112.63	140.64	126.64
LC	تفاؤل	Optimism	36.28	35.75	36.01	–11.18	33.5	11.16	138.65	136.53	137.59
LC	راحة	Rest	36.28	35.75	36.01	6.51	11.8	9.15	154.31	172.28	163.29
LC	سعادة	Happiness	36.28	35.75	36.01	–42.86	–9.58	–26.22	153.59	140.47	147.03
DC	زرعش	Grass	35.36	14.44	24.90	–12.82	–75.28	–44.05	83.31	90.89	87.10
DC	علم السعودية	Saudi Arabia flag	35.36	14.44	24.90	25.39	–81.44	–28.02	134.84	115.36	125.10
DC	أشجار	Trees	35.36	14.44	24.90	18.08	–46.76	–14.34	83.12	119.21	101.16
DC	كئابة	Depression	35.36	14.44	24.90	–42.14	–67.86	–55.00	–109.18	–135.30	–122.24
DC	ظلام	Darkness	35.36	14.44	24.90	–72.33	–113.02	–92.68	38.43	16.85	27.64
SB	بحر	Sea	69.3	45.33	57.31	136.29	123.02	129.66	104.71	135.38	120.05
SB	سماء	Sky	69.3	45.33	57.31	126.37	112.22	119.30	133.67	172.49	153.08
SB	ماء	Water	69.3	45.33	57.31	92.25	90.68	91.47	159.90	169.32	164.61
SB	الهدوء	Calm	69.3	45.33	57.31	31.61	41.78	36.69	163.78	150.00	156.89
SB	الراحة	Rest	69.3	45.33	57.31	33.49	51.66	42.58	143.88	161.55	152.71
SB	مسبح	Pool	69.3	45.33	57.31	112.43	138.4	125.42	72.78	110.53	91.65
SB	الصفاء	Serenity	69.3	45.33	57.31	41.14	57.32	49.23	117.14	141.64	129.39
SB	هلال	Crescent	69.3	45.33	57.31	36.76	–10.74	13.01	97.82	64.68	81.25
SB	النقاء	Purity	69.3	45.33	57.31	34.08	71.8	52.94	135.43	157.28	146.36
SB	نهر	River	69.3	45.33	57.31	63.84	97.4	80.62	111.47	124.87	118.17
SB	استرخاء	Rest	69.3	45.33	57.31	44.12	69.6	56.86	154.31	172.28	163.29
SB	الفرح	Happiness	69.3	45.33	57.31	–3.53	19.32	7.90	162.69	167.09	164.89
SB	المطر	Rain	69.3	45.33	57.31	55.39	108.22	81.81	142.27	172.13	157.20
LB	سماء	Sky	49.32	76.49	62.91	121.51	103.4	112.45	133.67	172.49	153.08
LB	بحر	Sea	49.32	76.49	62.91	98.86	65.7	82.28	104.71	135.38	120.05
LB	ماء	Water	49.32	76.49	62.91	93.25	60.1	76.68	159.90	169.32	164.61
LB	نهر	River	49.32	76.49	62.91	76.57	93.08	84.82	111.47	124.87	118.17
LB	الصفاء	Serenity	49.32	76.49	62.91	57.24	81.58	69.41	117.14	141.64	129.39
LB	شاطيء	Beach	49.32	76.49	62.91	46.59	34.68	40.63	112.63	140.64	126.64
LB	راحة	Rest	49.32	76.49	62.91	58.10	107.88	82.99	154.31	172.28	163.29
LB	هاديء	Calm	49.32	76.49	62.91	54.65	92.7	73.67	148.12	136.23	142.17
LB	غيوم	Clouds	49.32	76.49	62.91	31.25	78	54.63	150.92	169.17	160.04
LB	أمطار	Rain	49.32	76.49	62.91	38.73	66.56	52.64	154.31	164.91	159.61
LB	بارد	Cold	49.32	76.49	62.91	73.94	103.48	88.71	79.88	106.68	93.28
DB	بحر	Sea	94.68	41.76	68.22	87.90	63.7	75.80	104.71	135.38	120.05
DB	سماء	Sky	94.68	41.76	68.22	41.92	–0.48	20.72	133.67	172.49	153.08
DB	الراحة	Rest	94.68	41.76	68.22	15.67	–39	–11.67	143.88	161.55	152.71
DB	محيط	Ocean	94.68	41.76	68.22	93.24	133.96	113.60	69.61	84.25	76.93
DB	عميق	Deep	94.68	41.76	68.22	83.73	141.5	112.61	39.65	40.62	40.14
DB	الليل	Night	94.68	41.76	68.22	–6.29	86.76	40.23	117.45	98.74	108.09
DB	هدوء	Calm	94.68	41.76	68.22	4.24	16.92	10.58	148.12	136.23	142.17
DB	استرخاء	Rest	94.68	41.76	68.22	–4.29	17.62	6.66	154.31	172.28	163.29
DB	الغموض	Mystery	94.68	41.76	68.22	–18.92	82.36	31.72	21.45	16.94	19.20
DB	ماء	Water	94.68	41.76	68.22	16.12	–9.6	3.26	159.90	169.32	164.61
DB	غيوم	Clouds	94.68	41.76	68.22	–49.96	–25.76	–37.86	150.92	169.17	160.04
SP	ورد	Flowers	–34.78	81.24	23.23	61.06	30.56	45.81	117.04	124.11	120.58
SP	الطفولة	Childhood	–34.78	81.24	23.23	–51.43	–70.74	–61.09	81.06	99.72	90.39
SP	زهر	Flower/Rose	–34.78	81.24	23.23	43.73	10.54	27.13	79.65	82.42	81.03
SP	عنب	Grape	–34.78	81.24	23.23	29.51	37.66	33.58	79.45	75.91	77.68
SP	ورد البنفس	violets	–34.78	81.24	23.23	78.84	95.74	87.29	58.10	71.89	64.99
SP	بادنجان	Eggplant	–34.78	81.24	23.23	34.59	68.34	51.46	–10.41	–9.72	–10.06
SP	ايسكريم	Ice cream	–34.78	81.24	23.23	–21.33	–45.9	–33.62	122.41	134.34	128.37
SP	مريح	Comfortable	–34.78	81.24	23.23	–43.45	–45.4	–44.43	138.14	162.09	150.12
SP	مناكير	Manicure	–34.78	81.24	23.23	37.45	90.1	63.78	–43.04	79.70	18.33
SP	أنثوي	Feminine	–34.78	81.24	23.23	79.61	32.88	56.24	–6.78	88.47	40.85
LP	ورد	Flowers	–54.1	59.69	2.80	47.53	71.6	59.56	117.04	124.11	120.58
LP	انثوي	Feminine	–54.1	59.69	2.80	82.82	85.38	84.10	–6.78	88.47	40.85
LP	راحة	Rest	–54.1	59.69	2.80	–20.75	–3.8	–12.27	143.06	174.53	158.79
LP	جمال	Beauty	–54.1	59.69	2.80	16.75	43.78	30.26	121.53	133.15	127.34
LP	روج	Rouge/Lipstick	–54.1	59.69	2.80	18.61	48.92	33.76	–21.55	86.75	32.60
LP	زهر	Flower/Rose	–54.1	59.69	2.80	34.76	84.46	59.61	79.65	82.42	81.03
LP	طفولي	Childish	–54.1	59.69	2.80	–37.43	45.32	3.94	30.06	84.30	57.18
LP	الهدوء	Calm	–54.1	59.69	2.80	–44.06	–50.04	–47.05	148.12	136.23	142.17
LP	ناعم	Smooth	–54.1	59.69	2.80	48.41	67.02	57.72	55.96	130.21	93.08
LP	لطيف	Nice	–54.1	59.69	2.80	42.71	80.68	61.69	145.94	146.81	146.38
LP	الراحة	Rest	–54.1	59.69	2.80	–22.27	3.72	–9.28	154.31	172.28	163.29
LP	مناكير	Manicure	–54.1	59.69	2.80	57.61	84.2	70.90	–43.04	79.70	18.33
DP	بادنجان	Eggplant	–34.52	41.47	3.48	96.37	100.02	98.20	–10.41	–9.72	–10.06
DP	توت	Berry	–34.52	41.47	3.48	58.27	48.92	53.60	102.02	84.96	93.49
DP	ورد	Flowers	–34.52	41.47	3.48	73.08	41.1	57.09	117.04	124.11	120.58
DP	عنب	Grape	–34.52	41.47	3.48	82.82	64.4	73.61	79.45	75.91	77.68
DP	أنثوي	Feminine	–34.52	41.47	3.48	39.41	24.68	32.05	–6.78	88.47	40.85
DP	ورد الخزامى	Lavender rose	–34.52	41.47	3.48	87.90	89.68	88.79	73.94	79.47	76.71
DP	روج	Rouge/Lipstick	–34.52	41.47	3.48	25.41	–42.34	–8.46	–21.55	86.75	32.60
DP	هاديء	Calm	–34.52	41.47	3.48	–19.73	–77.36	–48.54	145.22	131.04	138.13
DP	مزعج	Annoying	–34.52	41.47	3.48	–26.67	–42.04	–34.35	–133.94	–113.45	–123.70

First, we present the analyses for the whole sample, and then we repeat the analyses for males and females separately. As this study aims to assess the predictive power of EVT, the critical analyses will be those which assess the correlation between WAVE and preference. For each of our three types of WAVE, the critical correlations which will indicate whether that WAVE model predicts color preference are those which pair the preference from one participant group (male, female or all) with the WAVE values derived from the responses of the corresponding group (e.g., T-WAVE_All_∼Preference_All_; T-WAVE_M_∼Preference_M_; T-WAVE_F_∼Preference_F_). This therefore requires a correction to the *p*-value for the three related comparisons to control for Type I error in our inference about the predictive value of each WAVE type. For these correlations we report Bonferroni-corrected *p*-values (divided by 3) using the notation p_corrected_. Other correlations and statistical analyses (e.g., cross-correlations of the WAVE from one sex group with the preference of the other) are conducted to enable the estimation and comparison of effect sizes and are reported with uncorrected *p*-values, which are not used for statistical inference.

### 3.1 Color preferences and weighted affective valence estimates for all participants

The 24 color stimuli were associated with 343 object terms, (males 157; females 186) and 412 concepts (males 168; females 244). Figure 1 gives the color preferences of the Saudi sample and the computed WAVES for their object (O-WAVE) or concept (C-WAVE) associations and all associations together (T-WAVE). Inspection of Figure 1 reveals that the Saudi color preference curve is highly similar to that described in Palmer and Schloss, with a minima at dark yellow that rises to a peak as hues become bluer. The WAVEs share some of these characteristics of the color preference curve, although the O-WAVE only accounts for 50% of the variance [*r*(22) = 0.71, p_corrected_ = 0.003], the C-WAVE 29% [*r*(22) = 0.54, p_corrected_ = 0.02], and the T-WAVE 44% [*r*(22) = 0.66, p_corrected_ = 0.002]. Statistical comparison of the magnitude of the correlations between preference and the different types of WAVE (using cocor–Diedenhofen and Musch, 2015) indicated that there was no significant difference between even the largest and smallest correlations [i.e., preference∼O-WAVE vs. preference∼C-WAVE, 95% confidence interval of the difference = (−0.1613, 0.5402) (Zou, 2007)]. A multiple linear regression with O-WAVE and C-WAVE as predictors, and color preference rating as the dependent variable was conducted to assess the contributions of object and concept associations together. The multiple regression model allows for these two components to vary in their relative weight, whereas the T-WAVE calculation treats objects and concepts equivalently. The model with both WAVE types together accounted for 59% of variance in color preference [*F*(2,21) = 15.16, *p* < 0.001, *r* = 0.77]. Both predictors were significant, with standardized beta coefficients of 0.59 (*p* < 0.001) for O-WAVE and 0.32 (*p* = 0.046) for C-Wave, indicating that O-WAVE was the stronger predictor. Interestingly, the variance explained for the multiple regression model was higher than that for the T-WAVE, which also incorporates both object and conceptual color associations.

### 3.2 Sex differences

**FIGURE 2:**
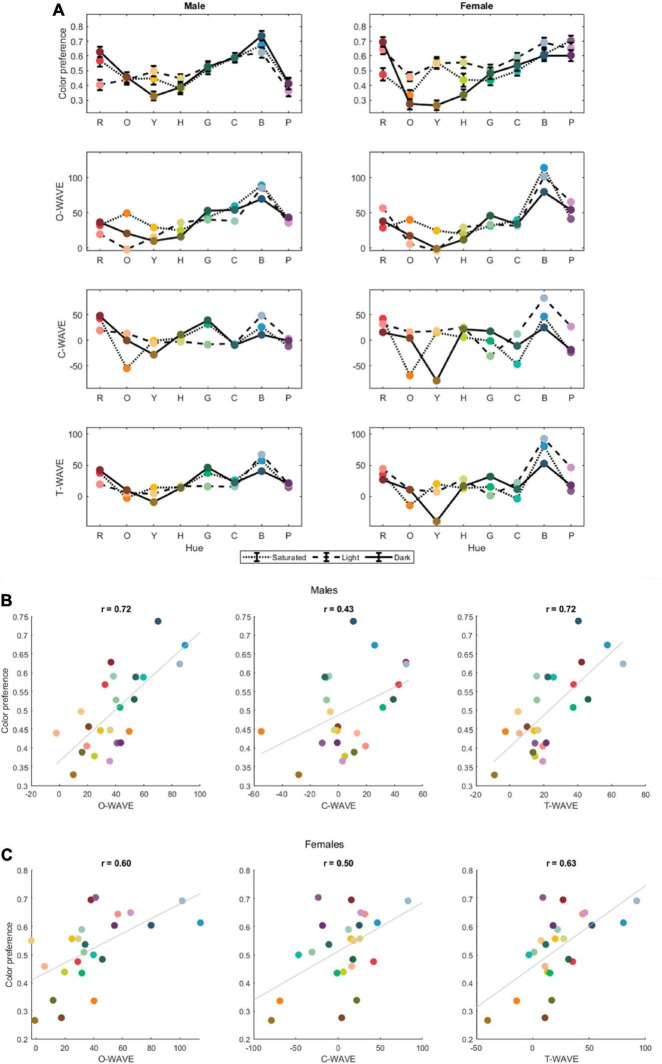
**(A)** The average color preference rating and WAVEs for saturated (S), light (L), dark (D) versions of 8 colors, for object associations (O-WAVE), concept associations (C-WAVE) and both together (T-WAVE), for male and female participants separately. **(B,C)** The correlation plots between color preference and the three WAVE types: O-Wave, C-Wave and T-Wave for male **(B)** and female participants **(C)**.

Figure 2 shows the color preference curves and three WAVEs for females and males separately. There are notable sex differences in the color preferences: it appears that males like purple less than females, and dark orange (brown) more, and female and male color preferences were not significantly correlated, [*r*(22) = 0.38, *p* = 0.07]. However, female and male WAVEs were correlated [O-WAVE *r*(22) = 0.86, *p* < 0.001; C-WAVE *r*(22) = 0.77, *p* < 0.001; T-WAVE *r*(22) = 0.83, *p* < 0.001].

**FIGURE 3:**
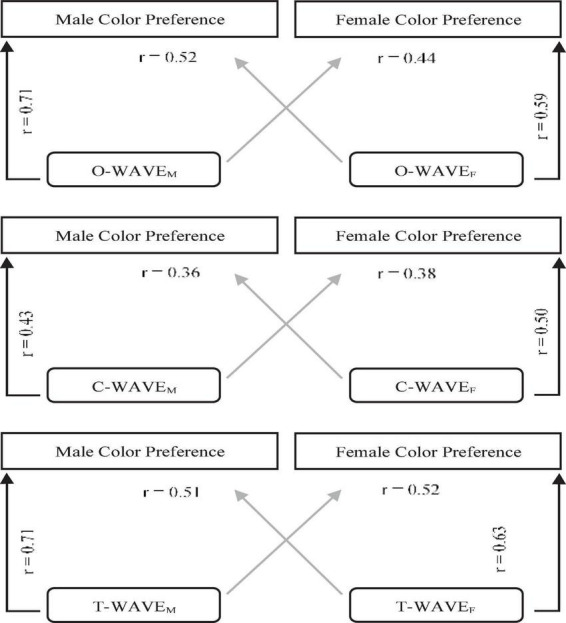
The correlation coefficient and Bonferroni-corrected *p*-values of shared variance between color preference and same-sex WAVEs (outer arrows), and color preference and opposite-sex WAVEs (inner diagonal arrows), for the three WAVE types (O-WAVE, C-WAVE, and T-WAVE).

Figure 3 gives the Pearson’s correlations correlating color preference with the same-sex WAVEs or the opposite sex WAVEs for all three WAVE types. As can be seen from the figure, more of the variance in preference was accounted for by the same-sex WAVES than the opposite sex WAVES for all three WAVE types, although the maximum amount of variance explained by same-sex WAVEs was only 50% for males (for the O-WAVE and the T-WAVE) and 40% for females (for the T-WAVE).

A multiple linear regression with O-WAVE_M_ and C-WAVE_M_ as predictors, and male color preference rating as the dependent variable was conducted to assess the contributions of object and concept associations for male participants. The model with both WAVE_M_ types together accounted for 57% of variance in color preference [*F*(2,21) = 13.89, *p* < 0.001, *r* = 0.76]. Both predictors were significant, with standardized beta coefficients of 0.64 (*p* < 0.001), for O-WAVE_M_ and 0.28 (*p* = 0.08) for C-WAVE_M_. For the female data, the model with both WAVE_F_ types together accounted for 42% of variance in color preference [*F*(2,21) = 7.51, *p* < 0.005, *r* = 0.65]. Only O-WAVE_F_ made a significant contribution to the model (standardized beta coefficient = 0.46, *p* < 0.05), while C-WAVE_F_ did not (standardized beta coefficient = 0.29, *p* = 0.14).

### 3.3 Number of associations

Taylor and Franklin (2012) found that for both their data and that of Palmer and Schloss (2010), the number of object associations also correlated with color preference. Here, there was no correlation between the number of associated objects and color preference for the total sample [*r*(22) = 0.04, *p* = 0.87], males [*r*(22) = 0.12, *p* = 0.59], or females [*r*(22) = 0.07, *p* = 0.74]. There was no significant correlation between the number of associated concepts and color preference for the total sample [*r*(22) = 0.46, *p*_corrected_ = 0.08], or for males [*r*(22) = 0.18, *p*_corrected_ > 0.999]. There was a significant correlation for the females [*r*(22) = 0.57, *p*_corrected_ = 0.012], although it is noteworthy that the variance in female color preference explained by number of listed concepts (32%) is less than that explained by the WAVE models reported above.

A multiple linear regression with number of object associations and number of concept associations as predictors, and color preference rating as the dependent variable was conducted. For the whole sample (men and women combined) the model with both predictors together accounted for 22% of variance in color preference [*F*(2,21) = 2.98, *p* = 0.07, *r* = 0.47]. Looking only at the male participants, the model with both predictors together accounted for 3% of variance in color preference [*F*(2,21) = 0.39, *p* = 0.68, *r* = 0.19]. In the female data the model with both number of object associations and number of conceptual associations together accounted for 37% of variance in color preference [*F*(2,21) = 6.05, *p* < 0.01, *r* = 0.61]. However, only the number of conceptual associations made a significant contribution to the model (standardized beta coefficient = 0.68, *p* < 0.005), while the number of object associations did not (standardized beta coefficient = −0.24, *p* = 0.23).

## 4 Discussion

Our analyses reveal that Saudi color preferences have some similarity to color preferences documented with similar stimuli for US [Palmer and Schloss (2010), Japanese (Yokosawa et al., 2016), and UK (Taylor and Franklin, 2012)] participants: in the current study Saudi preferences demonstrate the “characteristic” curve with a minimum of preference for dark yellow that rises to a peak for blue hues. For Saudi participants, the valence of the objects associated with the colors significantly accounted for color preference, but only for half of the variance. EVT therefore accounts for color preference less well for Saudi participants than for US (80%, Palmer and Schloss, 2010) or UK (66%, Taylor and Franklin, 2012) participants, but more so than Japanese (37%, Yokosawa et al., 2016) and Himba (23%, Taylor et al., 2013). We therefore provide some support for the generalisability of Ecological Valence Theory (the theory significantly accounted for Saudi color preference), although the findings also suggest that EVT is less successful at accounting for the color preference of some cultures than others.

Exactly why the predictive power of EVT appears culturally-dependent is unclear. The WAVE model already accounts for culturally specific color associations and their strength (e.g., “Saudi Arabian flag,” “sand,” “dust,” “palm tree trunk” are some of the cultural/environment-specific associations from the present study–see Table 2). It is not likely that the average strength of associations is a strong cultural factor as this would result in relatively flat WAVE curves, which are not observed in this study or others. However, it is possible that those cultures for which WAVE does not predict preference as well have greater individual variation in responses to the separate WAVE tasks (i.e., color preference rating, object description, object valence, object-color association rating). If individuals tend to differ more from one another in their responses to these tasks then the WAVE prediction will weaken. There is some evidence of this in the present study in that the gender-specific WAVE tended to fit gender-specific preference slightly better than the gender-crossed WAVE (e.g., male preference predicted by female WAVE, see Figure 2). Therefore, in cultures where people are more heterogeneous in their associations and ratings the WAVE may explain less variance.

Nevertheless, it is noteworthy that even for the Himba (Taylor et al., 2013) –where WAVE is a weaker predictor of color preference than found elsewhere–the variance explained is still substantial and significant. Therefore, the associations that people make between colors and objects appears to be a common component of color preference across cultures, but the weight of that component is culturally determined. Understanding how culture influences, as well as the other components of color preference is an important direction for future research.

We also examined sex differences in Saudi color preferences and how well EVT could account for female and male color preferences separately. There were notable differences in the female and male color preferences and in fact they did not correlate: females had a strong preference for purple and a strong dislike or dark orange (brown) that was lacking for males. A prior study of color preference in Saudi participants that used a set of iso-luminant and iso-saturated hues, found more pronounced sex differences in Saudi color preference than we do here: Al-Rasheed (2015) found a strong preference peak for purple for females that was absent for males and a strong preference peak for blue-green for males that was absent for females. Although in the current study male and female color preference did not correlate, the valence of color associations for males and females did, potentially suggesting a similar emotional response to color for males and females. The valence of object-color associations also did significantly correlate with color preferences for both males and females separately, but accounted for only 50% of the variance for males and 35% for females. Taylor and Franklin (2012) also similarly found that EVT accounted for male color preferences better than for females, but for a UK sample. Nevertheless, for the current study, same-sex correlations of preference and the valence of color associations accounted for more of the variance than opposite-sex correlations. These findings suggest that EVT does account for both Saudi female and male color preferences and that there is a degree of sex-specificity in the relationship between preference and the valence of color associations, although again no more than half of the variance is explained for either group. We also considered whether the biological mechanisms model of Hurlbert and Ling (2007) could account for preference any better and found it explained relatively little variance (20% females; 25% males) and did not outperform the WAVE model. This is consistent with prior work showing that this model does not predict color preference as well when the stimulus set varies in hue, lightness and saturation (Taylor et al., 2013)–Hurlbert and Ling’s model accounts for more variance in Saudi color preference for a stimulus set that varies only in hue (Al-Rasheed, 2015).

As an extension of prior published investigations of Ecological Valence Theory, the current study also measured abstract concept associations with the colors. We were surprised to see that participants actually offered more abstract concept than object associations, indicating that color is strongly conceptual as well as object based. However, the valence of concept associations did not significantly predict color preferences for Saudi participants, or for males or females separately. Adding concept associations to the WAVE with object associations also did not explain any more of the variance than object associations alone for Saudi participants or males, and only marginally more for females. These findings indicate that the valence of concept associations with color does not account for color preferences, at least for a Saudi sample. These findings fail to support a previously untested hypothesis proposed by Taylor and Franklin (2012), that adding concept associations might better predict color preferences particularly for females. However, in the current study, the number of concept associations did significantly correlate with only female color preference, although only accounting for just over a quarter of the variance. This provides tentative support for this notion that concept associations are related to color preference, but that it is the number of associations rather than the valence that contributes. Taylor and Franklin (2012) previously revealed that the number of object associations predicted both UK (66% shared variance) and US (80% shared variance) color preferences. However, we fail to find that relationship for Saudi color preferences, finding that only the number of concepts and not object associations correlates.

Overall, the findings of the current study suggest that degree to which the valence and number of object or abstract concept color associations accounts for color preferences varies across cultures: for Saudi color preference no more than half of the variance can be explained. We can see no differences in the method or procedure across studies that could account for why color preferences are less well explained by color associations for Saudi participants than the US participants in Palmer and Schloss’ original study. One possibility is that there are cultural reasons for the difference: that the factors that contribute to color preference vary across cultures as well as the color preferences themselves. Our findings highlight the importance of testing the generalisability of psychological theories across cultures, and not limiting psychological research to “WEIRD” populations (Western, Educated, Industrialized, Rich, and Democratic Henrich et al., 2010). Of course, with no more than half of the variance in Saudi color preference accounted for by EVT, the hunt for a model which can more fully account for why people like some colors more than others continues.

## Data availability statement

The original contributions presented in this study are included in the article/supplementary material, further inquiries can be directed to the corresponding author.

## Ethics statement

The studies involving human participants were reviewed and approved by the King Saud University, Deanship of Postgraduate Studies. The participants provided their written informed consent to participate in this study, which was conducted in accordance with the declaration of Helsinki.

## Author contributions

AA-R: involved in running the experiments and collecting the data. JM: involved in designing the software of the experiments and finding the colors of the stimulus. AA-R and JM: performed the analysis. AF: involved in planning and supervising the work. All authors reviewed the results and approved the final version of the manuscript.
